# Rational Design of Zeolites to Remove Siloxane-Related Pollutants with High Adsorption Loading and Enhanced Adsorption Energy

**DOI:** 10.1021/acsomega.4c10886

**Published:** 2025-06-06

**Authors:** Shiru Lin, Biao Liu, Yekun Wang, Yinghe Zhao, Arturo J. Hernández-Maldonado, Zhongfang Chen

**Affiliations:** † Department of Chemistry, 2310 University of Puerto Rico, Río Piedras, San Juan, Puerto Rico 00931, United States; ‡ Division of Chemistry and Biochemistry, Texas Woman’s University, Denton, Texas 76204, United States; § Department of Mathematics, 3404 University of North Texas, Denton, Texas 76203, United States; ∥ Department of Chemical Engineering, University of Puerto Rico, Mayagüez Campus, Mayagüez, Puerto Rico 00681, United States

## Abstract

Though siloxanes and their derivatives have been widely used, they are emerging and persistent pollutants in water systems. Developing high-performance and low-cost adsorbents to remove siloxane-related pollutants is an essential strategy for removing these contaminants. Through Grand Canonical Monte Carlo (GCMC) simulations, we computed and evaluated the adsorption performances of 246 experimentally available zeolite frameworks toward three silanols, namely, trimethylsilanol (TMS), dimethylsilanediol (DMSD), monomethylsilanetriol (MMST), and the coexisting contaminant in siloxane-impacted environments, dimethylsulfone (DMSO_2_), and obtained the best sorbents for each pollutant. To seek multifunctional zeolites, we first screened out the top 10 zeolite frameworks based on the loading values, among which the framework RWY showed the best performance. We further demonstrated that introducing dopants can enhance adsorption performance by taking RWY as an example. This work not only identified the most promising zeolite frameworks for removing linear siloxanes and derivatives, but also provided a relatively efficient and practical computational approach for screening sorbent materials for other emerging pollutants, balancing accuracy with tractable computational cost.

## Graphical abstract

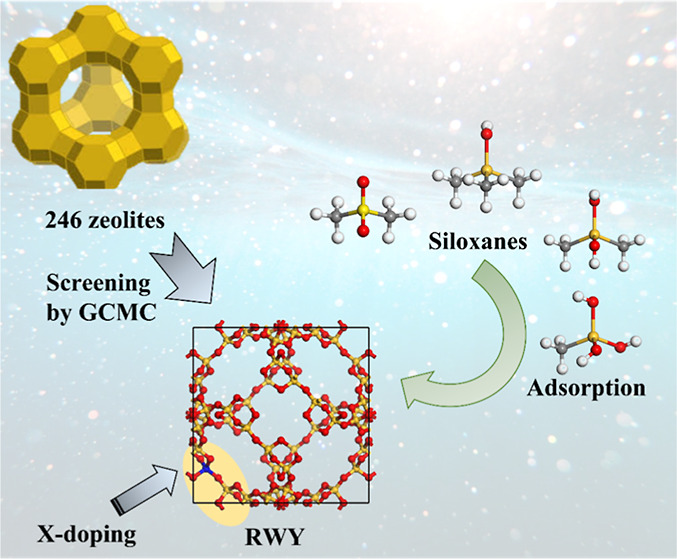

## 1 Introduction

As a class of critical organic compounds, siloxanes and their related compounds^1–7^ are widely used in many industries and products of daily use, including oil production,^8^ dry cleaning, personal care,^9^ and the manufacturing of high-weight silicon polymers.^5,10–12^ Siloxanes contain methyl substituents bonded to the silicon atoms of an alternating silicon–oxygen backbone and can be cyclic or linear in form.^13^ Due to their high vapor pressure^14^ and persistence to bioaccumulation,^15–20^ siloxanes and associated compounds have become emerging organic pollutants in water systems. Among others, The release of siloxanes and related compounds has severe potential toxic effects, for instance, connective tissue disease, adverse immunologic effects, and eventually fatal liver or lung damage on exposed animals.^9,21^ More critically, siloxanes could mask other pollutants in the detection systems, which hinders the effective removal of different contaminants.

Developing suitable sorbents is a cost-effective solution^22,23^ to the removal of siloxanes and related pollutants.^24–27^ Various adsorbents, such as ion exchange resins^28,29^ and activated carbon,^11,30,31^ have been explored. However, their adsorption abilities are far from satisfactory due to their low affinity for these pollutants.^32^ Therefore, high-performance and low-cost adsorbents are highly desired for removing siloxanes effectively.^28^

Zeolites are widely utilized for the adsorption of pollutants and drugs in aqueous systems^33–38^ due to their porous structures, remarkable thermal stability, and cost-effectiveness.^39–41^ To date, 246 zeolite framework types have been experimentally approved and can be obtained from the Database of Zeolite Structures by the International Zeolite Association Structure Commission (IZA-SC).^42^ Note that Hernandez-Maldonado and co-workers recently developed a hierarchical composite based on the confined space synthesis of a zeolite-type FAU within the pores of activated carbon that exhibits exceptional siloxane loadings of up to 20 mg cm^–3^ (or 17 mg g^–1^) from water, at least an order of magnitude larger compared to commercial carbon and resins.^43^ Very recently, they found that faulted UTD-1 pure silica zeolites with a DON-type framework to be highly effective in removing siloxanes from water. Especially, these zeolites have the highest adsorption capacity for TMS, reaching 73.1 mg/g in the 1–140 mg/L aqueous concentration range.^44^ To the best of our knowledge, these represent the only published studies utilizing zeolites in the liquid phase for siloxane removal.

Herein, we adopted a two-step strategy to screen and modify experimentally feasible zeolites to obtain high-adsorption-loading and high-adsorption-energy zeolites for the removal of siloxane-related contaminants. Our study focused on four representative compounds: trimethylsilanol (TMS), dimethylsilanediol (DMSD),^45,46^ and monomethylsilanetriol (MMST),^47^ which are the smallest silanol-containing products^48,49^ commonly found after the hydrolysis and sulphuration of longer linear and cyclic siloxanes, as well as dimethylsulfone (DMSO_2_)^45,49^ a structurally unrelated but environmentally relevant containment commonly detected alongside siloxanes in reclaimed water. Despite their environmental persistence, the removal of these silanol derivatives and associated contaminants remains challenging due to their low affinity for conventional adsorbents.

First, by high-throughput Grand Canonical Monte Carlo (GCMC) simulations, we screened out the top 10 sorbents for each pollutant among all the experimentally available zeolite frameworks (246 in total). Then, to achieve multifunctional zeolites, we screened out the top 10 zeolite frameworks with the largest loading value (with RWY as the best), and further showed the possibility of using doping atom (X) to improve the adsorption energy using RWY as an example. Besides the theoretically predicted high-performance sorbents toward siloxanes and derivatives, our screening and modification strategy provides a simple and fast approach to identity zeolite materials with both large loading capacity and high adsorption energies.

## 2 Computational Methods

Grand Canonical Monte Carlo (GCMC) simulations in the Sorption module of Materials Studio 2017 were employed to compute the absorption performance of 246 zeolite frameworks and six doped zeolites with each of four siloxane-related compounds. Fixed loading computations for RWY were performed by GCMC to find a suitable doping position for X-RWY under the same level of theory. The zeolite frameworks were treated as rigid in GCMC simulations using the sorption module.

GCMC is a statistical simulation that evaluates adsorption processes using random sampling and probabilistic interpretation in the sorbent framework. We calculated the average adsorption loading (mol nm^–3^), adsorption energy (kcal mol^–1^), and the 10 lowest-energy geometries of each adsorption system. Note that a higher loading and greater adsorption energy indicate a higher adsorption capacity for a sorbent. Fixed pressure adsorption simulations were carried out at a temperature of 298 K and a pressure of 101.33 kPa with Metropolis Monte Carlo method^50^ and COMPASS force field.^51,52^ Note that the COMPASS force field included the necessary parameters for both zeolites and siloxanes, and its validation was documented in the original paper.^51^ The reliability of the COMPASS force field has also been successfully demonstrated in simulating molecules similar to siloxanes, such as methacryloyloxymethyl)­dimethylethoxysilane, as well as polysiloxanes.^53,54^ Moreover, the COMPASS force field has been applied to simulate zeolites in various studies, including separating methane from carbon dioxide^55^ and methane from nitrogen.^56^

GCMC simulations have been widely used as a powerful tool to simulate the adsorption of gas molecules in porous materials such as zeolites.^57,58^ Since our primary purpose is to identify the most promising zeolite framework for the effective removal of siloxanes, we chose the uniform pressure, 101.313 kPa (atmospheric pressure), to approximate the experimental conditions when incorporating hydrophobic carbon materials with zeolites in adsorbing siloxanes.

In all the GCMC simulations, electrostatic interactions were treated using the Ewald summation method with a precision of 0.0001 kcal/mol and a 15.5 Å cutoff. van der Waals interactions were modeled with an atom-based scheme using cubic spline truncation and the same cutoff. To ensure reliable GCMC simulation results, each simulation comprised 6 × 10^6^ equilibration and 6 × 10^6^ production steps, with sampling every 25 steps. Monte Carlo moves included translation, rotation, regrowth, and exchange, using standard settings. The framework was treated as rigid and uncharged.

The initial zeolite structures were obtained from the IZA database. The Monte Carlo movements, including insertion, rotation, translation, reinsertion, and deletion, were randomly selected in each cycle. To eliminate the boundary effect, periodic boundary conditions were applied in all structures. We set 31.0 Å (15.5 Å cutoff distance) as a standard of the minimum length of a zeolite framework supercell to satisfy the minimum image convention. For example, the unit cell of RWY has *a* = *b* = *c* = 18.50 Å, therefore we use a 2 × 2 × 2 supercell of RWY, which has *a* = *b* = *c* = 37.00 Å.

Electrostatic interactions were treated using the Ewald summation method with a precision of 0.0001 kcal/mol and a 15.5 Å cutoff. van der Waals interactions were modeled with an atom-based scheme using cubic spline truncation and the same cutoff. To ensure reliable GCMC simulation results, each simulation comprised 6 × 10^6^ equilibration and 6 × 10^6^ production steps, with sampling every 25 steps. Monte Carlo moves included translation, rotation, regrowth, and exchange, using standard settings. The framework was treated as rigid and uncharged.”

DFT computations were done to optimize the four pollutants’ geometric structures and the metal-doped zeolite frameworks using the DMol^3^ code.^59,60^ The Perdew, Burke, and Ernzerhof (PBE) functional^61^ within a generalized gradient approximation (GGA) was used to describe the exchange–correlation potential. The density functional semicore pseudopotential (DSPP) was adopted for the relativistic effects of metal atoms, in which the core electrons are replaced by a single effective potential and some degree of relativistic corrections are introduced into the core,^62^ while the double numerical plus polarization (DNP) was chosen as the basis set for other elements. To maintain charge balance in tetravalent Al, Sc, or Zn doping, we introduced hydrogen atoms. For trivalent metals (Al and Sc), a single hydrogen atom was added to one of the four surrounding oxygen atoms, whereas for bivalent Zn, two hydrogen atoms were added to two of these oxygen atoms. Self-consistent field (SCF) computations were performed with a convergence criterion of 10^–5^ a.u. on the total energy and electronic computations.^61,71,72^

## 3 Results and Discussion

### 3.1 The Adsorption Performance of 246 Zeolite Framework for Siloxanes

We performed GCMC simulations to obtain the average adsorption loading and adsorption energy for each of the 246 zeolite frameworks toward each target pollutant molecule (Table S1). To visually show the distribution of the data set, we used the boxplots (Figure 1) to present the calculational results. The boxplot illustrates the distribution range of data, where each line on the box demarcates a section containing 25% of the data (a quartile). Thus, the first quartile, median, and third quartile are shown by the box’s bottom, middle, and top lines, respectively. Outliers are depicted as individual points. Moreover, if the notches of the two boxes do not overlap, it is evidence that the two medians differ.^63^

**Figure 1:**
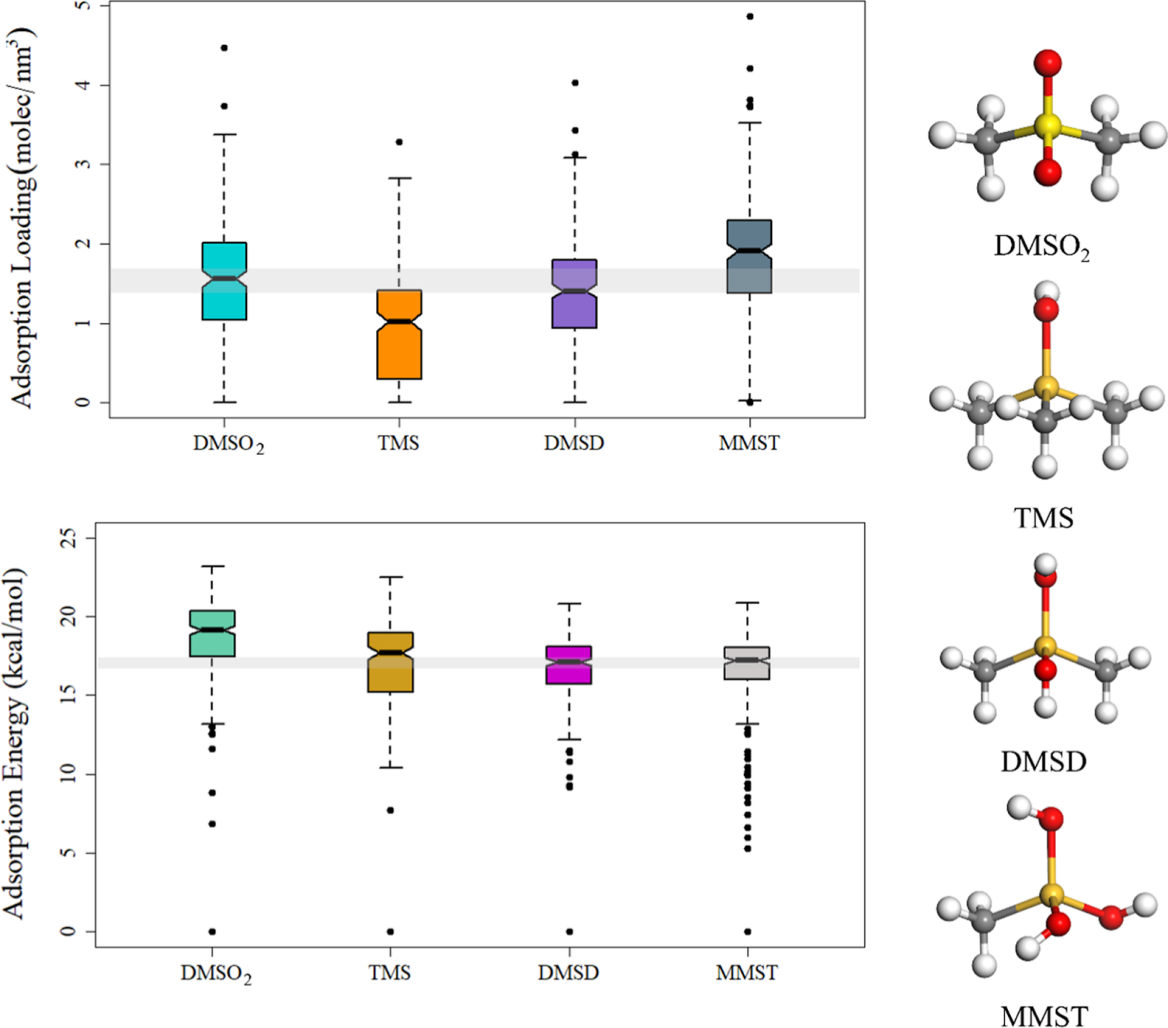
Box plots for adsorption loading and adsorption energy of 246 zeolite frameworks toward four siloxane-related compounds (left); geometries of four linear siloxanes and derivatives (right), where the gray, red, white, light yellow (only in DMSO_2_), and dark yellow balls stand for carbon, oxygen, hydrogen, sulfur, and silicon atoms.

The distribution ranges in the adsorption loading capacity boxplot (Figure 1) are quite large: 0 ∼ 3.5 mol nm^–3^ for DMSO_2_ and MMST, and 0 ∼ 3 mol nm^–3^ for TMS and DMSD. Note that the adsorption loading capacity of the best zeolite framework is three to four times higher than that of the worst framework (herein, the best and worst are evaluated only by the loading capacity). Thus, selecting suitable zeolite framework types is crucial for good adsorption loading. The medians for all four pollutants have similar values at around 1 ∼ 1.8. However, TMS has relatively small adsorption loading values: 50% of zeolite frameworks can only adsorb TMS with a loading value lower than 1, demonstrating that TMS is a sensitive pollutant toward the zeolite structure among four pollutants. The same phenomenon was observed for TMS adsorption in hypothetical zeolites^38^ and could be understood by its larger molecular size compared to other pollutant molecules. Moreover, the notches of DMSO_2_ and MMST boxes partially overlap, meaning that these two data sets have similar distributions.

From the adsorption energy boxplot (Figure 1), we found that TMS still has the broadest range of distribution; the differences between general maximum and minimal adsorption energy values for TMS are more than 10 kcal mol^–1^, while those of the other three pollutants are less than 10 kcal mol^–1^ (excluding extreme data points). DMSO_2_ has the highest median value (19.2 kcal mol^–1^) among all four pollutants, while TMS, DMSD, and MMST have close median values of 17.7, 17.1, and 17.2, respectively. Moreover, the down notches of TMS and the middle notches of DMSD and MMST have partial overlap, which means that these parts of the data may have similar distributions.

Considering the different distribution characteristics of four siloxane-related compounds for the zeolite framework, we first investigated the best zeolite framework for each pollutant. Since the adsorption energies are in the physisorption range (<30 kJ/Mol), we ranked the sorbents by the adsorption loading values to evaluate the performance of the 246 zeolite frameworks. The zeolite with the largest adsorption loading is the best for the specific pollutant (Table 1).

**Table 1:** Adsorption Loading (*E*_L_, mol nm^–3^) of the Top 10 Zeolite Frameworks for Each Linear Siloxane and Derivative^a^

	zeolite frameworks	*E*_L_	*E*_ad_		zeolite Frameworks	*E*_L_	*E*_ad_
DMSO_2_	RWY	4.5	16.7	TMS	RWY	3.3	14.7
	IRY	3.7	17.5		IRY	2.8	16.2
	IRR	3.4	17.2		IRR	2.7	16.4
	ITV	3.4	15.9		ITV	2.6	14.6
	ITT	3.3	18.0		ITT	2.5	17.1
	CLO	3.2	16.9		CLO	2.4	15.9
	JSR	3.2	18.6		IFU	2.3	16.1
	IFU	3.1	17.4		IFT	2.3	15.5
	IFT	3.0	16.8		SYT	2.2	16.9
	MEI	2.9	20.2		SAO	2.1	18.0
DMSD	RWY	4.0	15.0	MMST	RWY	4.9	17.1
	IRY	3.4	16.5		IRY	4.2	18.3
	IRR	3.1	16.2		IRR	3.8	17.8
	ITT	3.1	17.2		JSR	3.7	17.8
	ITV	3.1	14.4		ITT	3.7	18.3
	CLO	2.8	15.2		ITV	3.7	16.0
	IFU	2.8	16.2		CLO	3.5	16.4
	IFT	2.7	15.3		IFU	3.5	17.5
	SOD	2.7	16.1		MEI	3.4	19.9
	IWS	2.7	17.9		EMT	3.4	17.4

a The corresponding adsorption energies (*E*_ad_, kcal/mol) are also given for reference.

Table 1 presents the top 10 zeolite frameworks for each pollutant. Among these top zeolite frameworks, MMST has the highest loading of 4.9 mol nm^–3^, while TMS has the lowest loading of 3.3 mol nm^–3^. RWY has the largest loading for each of the four pollutants. The top six zeolites for adsorbing DMSO_2_, TMS, and DMSD are the same, in order with RWY, IRY, IRR, ITV, ITT, and CLO, respectively. Meanwhile, the seventh and eighth zeolites for DMSD and TMS are the same: IFU and IFT. For MMST, the top five zeolite frameworks are consistent with the other three siloxanes and derivatives. In addition, for MMST, JSR, ITT, and ITV have the same loading of 3.7 mol nm^–3^, and are tied for the fourth place. In terms of adsorption energies, DMSO_2_ adsorption on zeolite MEI exhibits the highest adsorption energy of 20.2 kcal/mol. However, this value is still well below the threshold for chemical adsorption.

### 3.2 Selecting High-Loading Zeolite Frameworks for Four Pollutants

The best zeolite frameworks for adsorption toward each of the four pollutants are different due to the variations in size, shape, and composition of the other harmful compounds. Note that the adsorption loading values for a pollutant are more related to the geometry type of zeolites, while the adsorption energy can be tuned, for example, by doping engineering on the frameworks. Therefore, to achieve multifunctional zeolites that work for the four siloxane-related compounds, we first used adsorption loading of the four pollutants to select the high-loading zeolite frameworks in this section, and then applied doping engineering to improve the adsorption energy of the best zeolite framework in Section 3.3.

Based on the aligned adsorption loading values for DMSO_2_ with TMS and DMSD with MMST (Figure 2), zeolite frameworks located in the upper right corners consistently exhibit high adsorption loadings for all four pollutants. A detailed review of both graphs with complete loading data reveals that the top 10 zeolites with the highest loadings out of 246 frameworks are RWY, IRY, IRR, ITT, ITV, CLO, IFU, IFT, JSR, and SBS (Table 2, Figure 3). Notably, RWY and IRY stand out with exceptional adsorption capacities, with RWY demonstrating the most significant loading values for all four pollutants.

**Figure 2:**
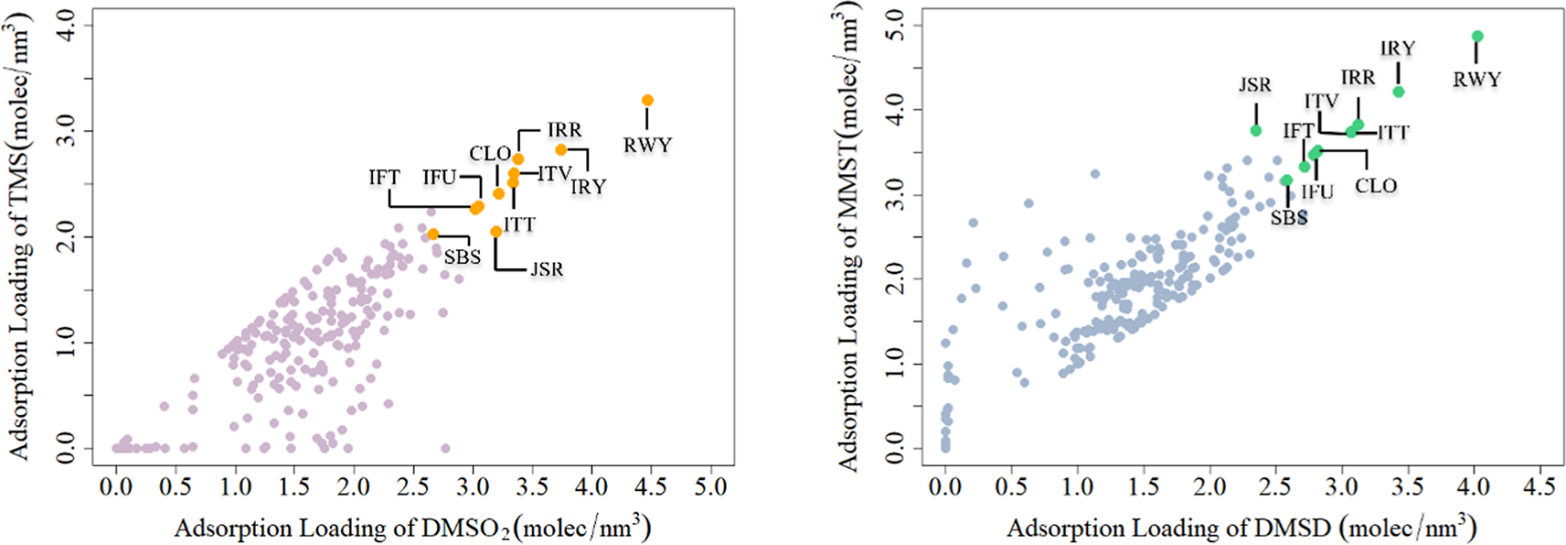
Distributions of adsorption loading for DMSO_2_ and TMS, as well as DMSD and MMST. The top 10 zeolite frameworks (based on loading values) are highlighted in orange (left) and green (right).

**Table 2:** Adsorption Loading Towards Four Siloxane-Related Compounds of the Top 10 Zeolite Frameworks Based on Loading Values From GCMC Simulations

adsorption loading (mol nm^–3^)				
zeolite frameworks	DMSO_2_	TMS	DMSD	MMST
RWY	4.5	3.3	4.0	4.9
IRY	3.7	2.8	3.4	4.2
IRR	3.4	2.7	3.1	3.8
ITT	3.3	2.5	3.1	3.7
ITV	3.3	2.6	3.1	3.7
CLO	3.2	2.4	2.8	3.5
IFU	3.1	2.3	2.8	3.5
IFT	3.0	2.3	2.7	3.3
JSR	3.2	2.1	2.4	3.8
SBS	2.7	2.0	2.6	3.2

**Figure 3:**
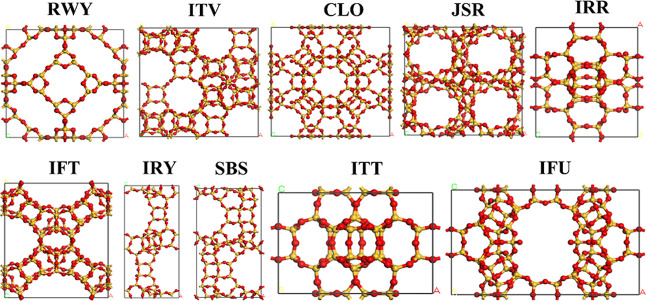
Unit cell structures of the top 10 zeolite frameworks selected based on the adsorption loading values toward DMSO_2_, TMS, DMSD, and MMST.

While the gallium–germanium-sulfide RWY zeolite has been successfully synthesized,^64^ the experimental realization of an all-silica RWY zeolite is yet to be achieved. However, despite this, exploring the potential of these yet-to-be-synthesized zeolites remains valuable for high-throughput screening and ensuring fair comparisons. Notably, investigations into all-silica RWY and other zeolite frameworks have been carried out using GCMC simulations to assess their potential in methane and carbon separation.^55^ Additionally, GCMC simulations confirmed that doping enhances benzene adsorption in NaY compared to all-silica Y zeolites, with results aligning well with experimental data.^33^

An RWY zeolite framework possesses I-43m symmetry, with the lattice parameters of the unit cell, *a* = *b* = *c* = 18.50 Å. Channels with diameters of about 5.12 Å are located around two types of open-hole structures, which have diameters of 9.05 Å and 3.77 Å, respectively (Figures 3 and S1). Note that RWY has the lowest framework density (7.6 T/1000 Å^3^) of the examined zeolite framework (in the range of 7.6 T/1000 Å^3^ to 18.0 T/1000 Å^3^), which likely explains the high guest loadings observed for this framework type. Generally, a lower framework density in zeolites can lead to larger pore volumes and greater pore accessibility, which can enhance the adsorption capacity, primarily due to the increased availability of adsorption sites and reduced steric hindrance.

Given the RWY framework’s superior loading values for all four compounds harmful to the environment relative to other zeolites, we chose to investigate the effect of doping on RWY. The subsequent section will detail the associated changes in adsorption energy and loading values.

### 3.3 Doping Engineering on the Highest-Loading Zeolite Framework RWY

Doping metals can transfer electrons to adsorbents to enhance adsorptions, which renders doping engineering an effective way to boost sorbents^33,65^ and catalysts.^66,67^ We chose six commonly used experimental dopants—Sn, Ge, Ti, Al, Sc, and Zn^67–71^ —to evaluate their impact on the adsorption performance of RWY toward the four pollutants.

Before investigating doping engineering, we ought to find the most suitable metal doping sites. To identify the best doping sites for RWY, we carried out the GCMC simulations using the Fixed Loading protocol in the Sorption module with loading = 1 to obtain the most stable adsorption geometries. Note that when the loading value is fixed to one, the lowest energy adsorption geometries present the most favorable adsorption site for a specific pollutant. Remarkably, DMSO_2_ and DMSD tend to be adsorbed in the bottom left corner, TMS prefers a position slightly to the right, and MMST tends to be adsorbed at a further right position, near the middle bottom of RWY (Figure 4). Most of the linear siloxanes and derivatives are adsorbed around the left and bottom channels, indicating that this area significantly influences adsorption performance. The specific adsorption sites for different siloxanes are primarily influenced by their ability to form hydrogen bonds with the RWY framework. All these four siloxane-related compounds establish hydrogen bonds between the hydrogen atoms of the siloxanes and the oxygen atoms of the RWY framework, with bond distances ranging from 1.7 Å to 3.0 Å. Based on these observations and considering the symmetry of the RWY framework, we chose the silicon vacancy close to the bottom left channel (Figure 4) as the site for doping engineering.

**Figure 4:**
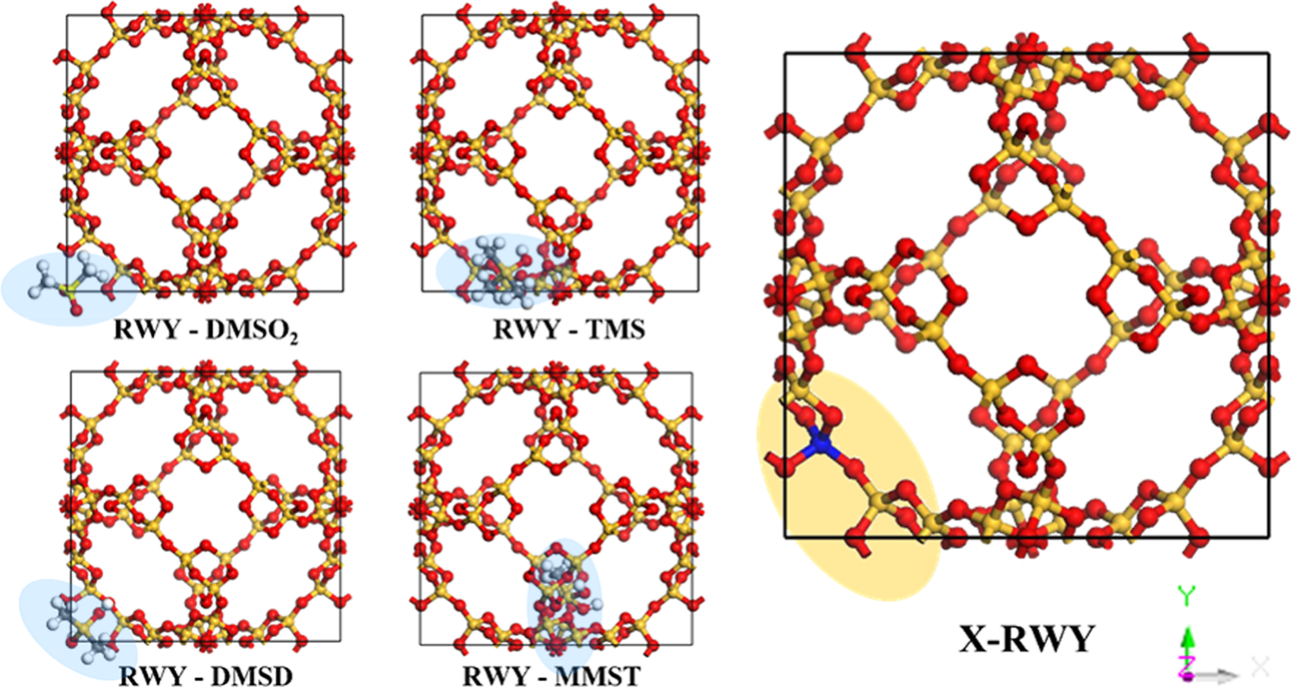
Most stable adsorbed geometries of RWY with four siloxane-related compounds (in blue shadows), and the structure of metal-doped RWY (X-RWY). Light yellow (visible only in DMSO_2_), yellow, red, white, and blue atoms represent sulfur, silicon, oxygen, hydrogen, and metal, respectively.

Upon attaining a favorable doping site, we optimized pristine RWY and six doped structures (denoted as X-RWY), computed their adsorption energies and loadings toward four pollutants by GCMC simulations, and then compared the adsorption performance before and after doping (Figure 5).

**Figure 5:**
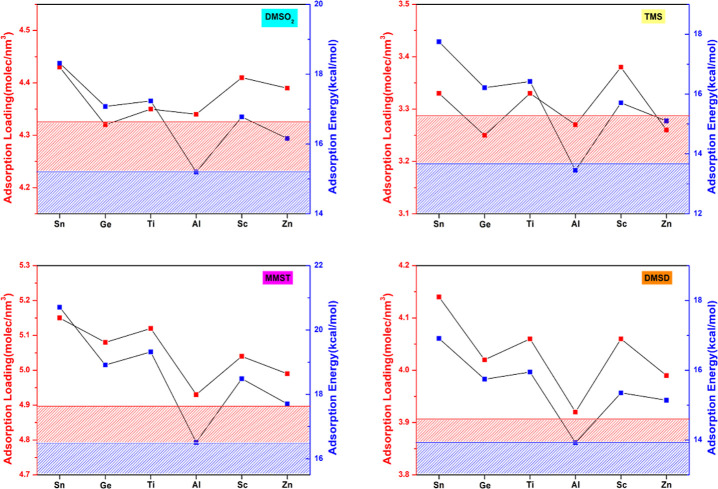
Adsorption energy and loading of six X-RWY zeolites toward four siloxane-associated pollutants, where the red lines represent the adsorption energy, blue lines represent the adsorption loading values, and the dash lines stand for the corresponding values of the pristine RWY zeolite framework.

After substituting a silicon atom in the RWY structure with a metal and performing DFT structural optimization, the bond lengths between the substituted metal and three coordinating oxygen atoms increase, accompanied by changes in bond angles. Among the six investigated metals, Ge and Ti exhibit minimal elongation, with bond length increases of less than 0.2 Å compared to the original RWY structure. In contrast, Sn doping results in significantly longer bond lengths, reaching approximately 2 Å with the coordinating oxygen atoms. For Al, Sc, and Zn doping, which require additional hydrogen for stabilization, the bond lengths between the metal and the hydrogen-bonded oxygen also extend to around 2 Å. The detailed comparisons are presented in Figures S2 and S3 in the Supporting Information.

Encouragingly, most X-RWY structures exhibit increased adsorption energies toward four pollutants except for Al, which has only a minimal influence. Doping engineering influences the TMS adsorption energies most significantly. Compared with pristine RWY, five doped X-RWY increase the adsorption energy toward TMS by 11.4% to 31.0%, giving an average 19.8% increase in adsorption energies. The improvement rate of adsorption loading toward MMST is the second highest, from 8.1% to 26.5%, giving an average 16.2% increase in adsorption energies. The improvement rate of adsorption energy toward DMSD is lower than that of MMST and TMS, which is from 9.6% to 22.4%, averaging an increase of 14.5%. Doping effects have less influence on X-RWY toward DMSO_2_, which still increases from 6.99% to 21.26%, averaging an increase of 13.3%.

Doping can maintain the high adsorption loading of the RWY framework well. The doped systems usually have higher adsorption loading than the pristine RWY. The vastly improved adsorption energy and stable adsorption loading demonstrate that doping can help achieve multifunctional zeolites with both high adsorption loading and high adsorption energy.

Why can doping increase the adsorption energy? It is because doping modifies the electronic environment of the zeolite framework due to the differing electronegativity values,^72^ atomic radii, and bond lengths of the doping atoms compared to silicon. For example, bond lengths vary significantly among common dopants (Si–O 1.62 Å, Sn–O 2.22 Å, Ge–O 1.79 Å, Ti–O 1.99 Å, Al–O 1.94 Å, Sc–O 3.06 Å, Zn–O 1.98 Å in our doped RWY), leading to changes in the local chemical environment near the adsorption sites. These changes can enhance the electrostatic interactions between the RWY framework and the siloxanes by altering the relative positions and electronic density around the adsorption sites. Our previous work^38^ revealed that electrostatic interactions are crucial for enhancing the performance of doped RWY, confirming the significance of dopants in the adsorption process.

Considering different doping atoms of RWY toward siloxanes, Sn-RWY always brings the most significant enhancement of adsorption energies compared with the pristine RWY. Sn-RWY increases adsorption energy by 21.3, 31.0, 22.4, and 26.5% for DMSO_2_, TMS, DMSD, and MMST, respectively. Ti and Ge rank second and third, respectively, in terms of improvements in adsorption energy. Al-RWY presents a slight decrease in adsorption energy toward TMS (−0.8%) and a slight increase toward the other three siloxanes. In the X-RWY series, Zn-RWY ranks second to last in improving adsorption energy toward four pollutants, while Sc-RWY is third to last.

To summarize, doping metals improve zeolite adsorption energy to siloxane-related compounds, provided that the adsorption loading does not change significantly. Sn-RWY has the most enhanced adsorption energy toward four siloxane-associated pollutants and even increases the adsorption loading of the RWY framework (Table 3). Thus, Sn- has a powerful ability to enhance the adsorption energy and loading of the RWY framework, rendering Sn-RWY promising sorbents to adsorb four linear siloxane-related pollutants efficiently. Al is the worst dopant for RWY toward adsorption performance due to the lowest change or decrease in adsorption energy among X-RWY. Thus, we rank the recommended dopants for RWY toward siloxanes as follows: Sn > Ti > Ge > Sc > Zn > Al.

**Table 3:** Adsorption Loading (mol nm^–3^) and Adsorption Energy (kcal mol^–1^) of Sn-RWY Toward DMSD, DMSO_2_, MMST, and TMS, as Determined by GCMC Simulations

adsorbates	adsorption loading	increase rate of loading (%)	adsorption energy	increase rate of loading (%)
DMSO_2_	4.4	2.6	18.3	21.3
TMS	3.3	1.2	17.8	31.0
DMSD	4.1	6.2	16.9	22.4
MMST	5.2	5.3	20.7	26.5

The above studies demonstrate a proof of concept that strategic doping can enhance adsorption strength while maintaining the loading capacity of pristine zeolites. We focused on doping inherently active and accessible adsorption sites, which are prime targets for improving adsorption characteristics. However, in principle, it is possible to create more adsorption sites by doping other areas, such as framework intersections or channel intersections, which could modify the framework’s internal surface area and electronic properties. This approach may lead to the formation of new adsorption sites or alter existing ones, potentially increasing the adsorbent’s capacity and selectivity. Future studies could explore this hypothesis by systematically doping various framework sites and evaluating the resultant adsorption properties.

## 4 Conclusion

In this work, we investigated the potential of 246 experimentally approved zeolite frameworks for the adsorption of four siloxane-related pollutants using GCMC simulations. Among 246 zeolite frameworks, RWY possesses the highest loading for all four pollutants. We further modified the highest-loading zeolite framework, RWY, by doping six dopants separately: Sn, Ge, Ti, Al, Sc, and Zn. Upon doping, we determined that Sn-RWY has the strongest adsorption energy for four siloxane-associated pollutants.

To summarize, computational screening methods provide a way to rapidly assess the potential of zeolite frameworks by modeling adsorption interactions, and the most promising materials can be further modified for improved performance. This computational study quickly narrowed down the list of zeolites with effective adsorption of DMSO_2_, TMS, DMSD, and MMST. It is noteworthy that, in contrast to previous studies that screened hypothetical pure-silica zeolites,^38^ this work computed and evaluated the adsorption performances of 246 experimentally available zeolite frameworks on four linear siloxane-related pollutants, which provides experimental peers with clear guidelines for developing high-performance sorbents. Moreover, this computational strategy can be broadly applicable as a blueprint for designing sorbents to remove other environmentally harmful pollutants.

## Supplementary Material



## Notes

The adsorption loading and adsorption energy of 246 zeolite frameworks toward DMSO_2_, TMS, DMSD, and MMST (PDF)
