# Supplementary material for: Rational Design of Zeolites to Remove Siloxane-Related Pollutants with High Adsorption Loading and Enhanced Adsorption Energy
Source: ACS Omega. 2025 Jun 6;10(23):24160–8. doi: 10.1021/acsomega.4c10886 (PMC12177750; doi:10.1021/acsomega.4c10886)
Supplement: Supplementary file 1 [file ao4c10886_si_001.pdf]

Rational Design of Zeolites to Remove Siloxane-Related Pollutants with High Adsorption Loading and Enhanced Adsorption Energy

Shiru Lin,[†1,2] Biao Liu, [†1] Yekun Wang,[3] Yinghe Zhao,[1]

Arturo J. Hernández-Maldonado,[4*] Zhongfang Chen[1*]

[1] Department of Chemistry, University of Puerto Rico, Río Piedras, San Juan, PR 00931, USA

[2] Division of Chemistry and Biochemistry, Texas Woman's University, Denton, Texas 76204, USA

[3] Department of Mathematics, University of North Texas, Denton, TX 76203, USA

[4] Department of Chemical Engineering, University of Puerto Rico, Mayagüez Campus, Mayagüez, PR 00681, USA

* To whom correspondence should be addressed. Email: arturoj.hernandez@upr.edu (A.H); zhongfang.chen1@upr.edu (Z.C.)

† These authors contributed equally to this work

Table S1. The adsorption loading (molec nm^{-3}) and adsorption energy (kcal mol^{-1}) of all the frameworks toward $DMSO_2$.

Zeolite Framework	Rank of Adsorption Loading	Adsorption Loading	Rank of Adsorption Energy	Adsorption Energy (kcal/mol)	Sum of Ranks
RWY	1	4.3	180	16.69	181
IRY	2	3.6	183	16.63	185
IRR	3	3.5	178	16.81	181
ITV	4	3.3	194	15.28	198
PUN	5	3.3	16	21.97	21
EDI	6	3.3	152	18.14	158
IFU	7	3.2	172	17.11	179
JSR	8	3.1	26	18.22	34
THO	9	3.1	147	21.23	156
CLO	10	3.1	190	16.12	200
SBS	11	3.1	123	18.9	134
ITT	12	3	188	16.2	200
MEI	13	3	53	20.2	66
LTA	14	3	104	19.19	118
OBW	15	3	115	19.06	130
EMT	16	2.9	163	17.6	179
SOD	17	2.8	7	22.68	24
EPI	18	2.8	17	21.83	35
SAO	19	2.7	139	18.42	158
NPT	20	2.7	71	19.8	91
EWT	21	2.7	138	18.43	159
AFY	22	2.6	106	19.16	128
BPH	23	2.6	105	19.17	128
SBT	24	2.6	166	17.47	190
ETR	25	2.6	63	19.95	88
BEC	26	2.5	97	19.31	123
SBE	27	2.5	173	17.08	200
ISV	28	2.5	114	19.07	142
FAU	29	2.5	185	16.4	214
DFO	30	2.5	121	18.93	151
VFI	31	2.4	175	16.91	206
UTL	32	2.4	128	18.61	160
POS	33	2.4	91	19.47	124
SAV	34	2.4	184	16.44	218
EAB	35	2.4	27	21.11	62
UFI	36	2.4	92	19.43	128
IWS	37	2.4	153	18.09	190

Zeolite Framework	Rank of Adsorption Loading	Adsorption Loading	Rank of Adsorption Energy	Adsorption Energy (kcal/mol)	Sum of Ranks
RHO	38	2.4	131	18.58	169
AFS	39	2.4	146	18.27	185
ATT	40	2.3	43	20.74	83
JST	41	2.3	58	20.11	99
BRE	42	2.3	11	22.37	53
AVL	43	2.3	37	20.86	80
ZON	44	2.3	4	23.2	48
_CON	45	2.2	93	19.43	138
BOG	46	2.2	85	19.52	131
IWR	47	2.2	20	19.37	67
JSN	48	2.2	95	21.57	143
ATN	49	2.2	9	22.52	58
WEN	50	2.2	90	19.48	140
BOZ	51	2.2	168	17.42	219
SFS	52	2.2	61	20	113
CHA	53	2.2	86	19.52	139
IFO	54	2.2	150	18.16	204
TSC	55	2.2	100	19.26	155
ITN	56	2.1	84	19.54	140
IFR	57	2.1	41	20.75	98
OWE	58	2.1	22	21.34	80
KFI	59	2.1	116	19.03	175
BEA	60	2.1	144	18.34	204
CGS	61	2.1	21	21.46	82
AFV	62	2.1	49	20.56	111
CGF	63	2.1	13	22.21	76
OSO	64	2.1	191	16.05	255
MEP	65	2.1	8	22.55	73
FRA	66	2.1	23	21.31	89
SFN	67	2.1	157	17.8	224
SEW	68	2.1	50	20.51	118
SAT	69	2	32	20.99	101
PHI	70	2	33	20.95	103
IWV	71	2	68	17.14	139
MSE	72	2	154	19.88	226
SFH	73	2	171	18.06	244
UOV	74	2	87	19.51	161
FAR	75	2	15	22	90
RUT	76	2	2	23.85	78
RTH	77	2	110	19.1	187
STW	78	2	83	19.59	161

Zeolite Framework	Rank of Adsorption Loading	Adsorption Loading	Rank of Adsorption Energy	Adsorption Energy (kcal/mol)	Sum of Ranks
MAR	79	2	10	22.41	89
ATS	80	2	145	18.31	225
CDO	81	2	5	22.96	86
UWY	82	2	89	19.48	171
FER	83	2	28	21.09	111
HEU	84	1.9	31	21.09	115
IWW	85	1.9	176	16.86	261
USI	86	1.9	122	18.92	208
IRN	87	1.9	124	18.77	211
SVV	88	1.9	3	23.63	91
UOZ	89	1.9	6	24.05	96
ASV	90	1.9	158	22.88	249
AFR	91	1.9	161	17.64	253
SFO	92	1.9	151	17.69	244
SAS	93	1.9	47	18.15	141
STI	94	1.9	137	20.62	232
AFT	95	1.8	126	18.43	222
AFX	96	1.8	40	18.67	137
ITH	97	1.8	96	20.77	194
OFF	98	1.8	80	19.35	179
ERI	99	1.8	18	19.61	118
GIU	100	1.8	46	21.74	147
ITR	101	1.8	69	20.7	171
ITG	102	1.8	77	19.67	180
MEL	103	1.8	76	19.82	180
MAZ	104	1.8	174	19.69	279
LEV	105	1.8	148	17.05	254
SFW	106	1.7	135	18.2	242
SSF	107	1.7	136	18.46	244
SSO	108	1.7	99	18.44	208
EON	109	1.6	56	19.26	166
TUN	110	1.6	72	20.19	183
STT	111	1.6	117	19.77	229
TER	112	1.6	34	19	147
LTN	113	1.6	109	20.92	223
SOF	114	1.6	181	19.13	296
SBN	115	1.6	155	16.67	271
CSV	116	1.6	132	17.99	249
MOZ	117	1.6	81	18.56	199
AST	118	1.6	127	19.61	246
NES	119	1.6	55	18.63	175

Zeolite Framework	Rank of Adsorption Loading	Adsorption Loading	Rank of Adsorption Energy	Adsorption Energy (kcal/mol)	Sum of Ranks
ATO	120	1.6	107	20.19	228
OKO	121	1.5	12	19.15	134
JSW	122	1.5	62	22.24	185
LTF	123	1.5	141	19.99	265
MWW	124	1.5	24	18.4	149
BOF	125	1.5	51	21.3	177
MTT	126	1.5	103	20.31	230
TON	127	1.5	65	19.2	193
SIV	128	1.5	44	19.92	173
LAU	129	1.5	59	20.72	189
JOZ	130	1.5	66	20.1	197
SVR	131	1.5	25	19.91	157
TOL	132	1.5	182	21.27	315
IFW	133	1.5	14	16.65	148
ESV	134	1.5	156	22.06	291
LIO	135	1.5	67	17.86	203
UOS	136	1.5	36	19.89	173
DAC	137	1.5	164	20.87	302
ITE	138	1.5	113	17.59	252
EZT	139	1.4	57	19.08	197
RTE	140	1.4	74	20.14	215
SFG	141	1.4	187	19.75	329
GIS	142	1.4	38	16.32	181
AFG	143	1.4	64	20.78	208
AWW	144	1.4	112	19.93	257
AFI	145	1.4	169	17.32	315
CAN	146	1.4	35	19.09	182
LOS	147	1.4	29	20.88	177
PON	148	1.4	111	21.09	260
EUO	149	1.4	101	19.09	251
IMF	150	1.4	75	19.24	226
MSO	151	1.3	177	19.73	329
AEI	152	1.3	179	16.85	332
GME	153	1.3	42	16.73	196
IHW	154	1.3	78	20.74	233
IFY	155	1.3	125	19.66	281
MTW	156	1.3	170	18.73	327
STF	157	1.3	189	17.24	347
SFE	158	1.2	186	16.16	345

Zeolite Framework	Rank of Adsorption Loading	Adsorption Loading	Rank of Adorption Energy	Adsorption Energy (kcal/mol)	Sum of Ranks
SSY	159	1.2	73	16.32	233
SAF	160	1.2	119	18.97	280
VET	161	1.2	94	19.75	256
GON	162	1.2	142	19.4	305
MFS	163	1.2	88	18.4	252
SOS	164	1.1	167	17.43	332
UEI	165	1.1	133	19.5	299
SFV	166	1.1	160	18.52	327
SFF	167	1.1	159	17.65	327
OSI	168	1.1	98	17.67	267
SGT	169	1.1	129	19.27	299
PAU	170	1.1	60	18.6	231
AFN	171	1.1	79	20	251
EEI	172	1.1	143	19.65	316
DON	173	1.1	196	18.37	370
CFI	174	1	118	15.1	293
MFI	175	1	120	19	296
MER	176	1	134	18.95	311
STO	177	1	192	18.5	370
AET	178	1	19	15.91	198
MTN	179	1	45	21.6	225
ETL	180	1	140	20.71	321
SZR	181	1	70	18.41	252
AFO	182	1	52	19.82	235
AEL	183	1	82	20.3	266
DOH	184	1	30	19.6	215
MTF	185	0.9	108	21.09	294
DDR	186	0.9	48	19.15	235
PCR	187	0.8	149	20.58	337
LTL	188	0.8	54	18.2	243
MRE	189	0.8	102	20.2	292
NON	190	0.8	130	19.23	321
MWF	191	0.8	193	18.58	385
MOR	192	0.7	165	15.82	358
RON	193	0.7	39	17.58	233
PSI	194	0.6	162	20.78	357
AWO	195	0.3	197	17.64	393
VNI	196	0.3	195	14.71	392
JRY	197	0.2	1	15.16	90
ABW	198	0	198	0	396
ACO	199	0	199	0	398

Zeolite Framework	Rank of Adsorption Loading	Adsorption Loading	Rank of Adorption Energy	Adsorption Energy (kcal/mol)	Sum of Ranks
AEN	200	0	200	0	400
AHT	201	0	201	0	402
ANA	202	0	202	0	404
APC	203	0	203	0	406
APD	204	0	204	0	408
ATV	205	0	205	0	410
BCT	206	0	206	0	412
BIK	207	0	207	0	414
BSV	208	0	208	0	416
CAS	209	0	209	0	418
CHI	210	0	210	0	420
CZP	211	0	211	0	422
DFT	212	0	212	0	424
GOO	213	0	213	0	426
ITW	214	0	214	0	428
JBW	215	0	215	0	430
JNT	216	0	216	0	432
LIT	217	0	217	0	434
LOV	218	0	218	0	436
LTJ	219	0	219	0	438
MON	220	0	220	0	440
MVY	221	0	221	0	442
NAB	222	0	222	0	444
NAT	223	0	223	0	446
NPO	224	0	224	0	448
NSI	225	0	225	0	450
PAR	226	0	226	0	452
RRO	227	0	227	0	454
RSN	228	0	228	0	456
RWR	229	0	229	0	458
VSV	230	0	230	0	460
WEI	231	0	231	0	462
YUG	232	0	232	0	464

Table S2. The adsorption loading (molec nm^{-3}) and adsorption energy (kcal mol^{-1}) of all the frameworks toward TMS.

Zeolite Framework	Rank of Adsorption Loading	Adsorption Loading	Rank of Adsorption Energy	Adsorption Energy (kcal/mol)	Sum of Rank
RWY	1	3.3	149	15.66	150
EDI	2	2.9	171	14.23	173
IRY	3	2.6	154	15.46	157
IRR	4	2.5	148	15.74	152
ITV	5	2.4	170	14.24	175
CLO	6	2.3	147	15.86	153
AVL	7	2.1	25	19.75	32
IFU	8	2.1	155	15.41	163
BPH	9	2.1	54	18.67	63
JSR	10	2	125	16.88	135
SAO	11	2	92	17.77	103
ITT	12	1.9	159	15.12	171
BEA	13	1.9	65	18.49	78
SBT	14	1.9	93	17.74	107
FRA	15	1.9	163	14.98	178
BEC	16	1.9	73	18.34	89
SBS	17	1.9	134	16.54	151
ISV	18	1.9	71	18.36	89
EWT	19	1.9	122	17.01	141
STI	20	1.9	6	21.52	26
ETR	21	1.8	63	18.55	84
POS	22	1.8	58	18.6	80
ERI	23	1.8	13	20.56	36
LEV	24	1.8	69	18.43	93
NPT	25	1.8	123	16.91	148
SFO	26	1.8	74	18.34	100
EMT	27	1.7	105	16.17	132
IWS	28	1.7	141	17.47	169
OBW	29	1.7	139	16.2	168
SBE	30	1.7	140	16.18	170
PUN	31	1.7	40	19.11	71
BOZ	32	1.7	107	17.43	139
IWR	33	1.7	60	18.58	93
MEI	34	1.7	90	17.84	124
FAU	35	1.6	150	15.66	185
_CON	36	1.6	47	18.85	83

Zeolite Framework	Rank of Adsorption Loading	Adsorption Loading	Rank of Adsorption Energy	Adsorption Energy (kcal/mol)	Sum of Rank
VFI	37	1.6	157	15.24	194
AST	38	1.6	9	21.22	47
ITN	39	1.6	62	18.57	101
AFV	40	1.6	15	20.34	55
AFS	41	1.6	109	17.35	150
KFI	42	1.6	64	18.55	106
DFO	43	1.6	110	17.34	153
OKO	44	1.5	17	20.14	61
UOV	45	1.5	42	19.08	87
USI	46	1.5	30	19.51	76
LAU	47	1.5	28	19.7	75
MWW	48	1.5	61	18.57	109
OSO	49	1.5	146	15.88	195
TSC	50	1.5	2	23.69	52
CGS	51	1.5	39	19.11	90
MSE	52	1.5	53	18.71	105
NES	53	1.5	35	19.34	88
ESV	54	1.4	3	22.21	57
EZT	55	1.4	34	19.37	89
OFF	56	1.4	76	18.32	132
RTE	57	1.4	8	21.27	65
EAB	58	1.4	24	19.86	82
AWW	59	1.4	7	21.34	66
CAN	60	1.4	166	14.75	226
LOS	61	1.4	26	19.72	87
SAT	62	1.4	38	19.17	100
AFG	63	1.4	57	18.6	120
PHI	64	1.4	119	17.06	183
ATS	65	1.3	127	16.77	192
MTT	66	1.3	113	17.23	179
UWY	67	1.3	121	17.03	188
MTW	68	1.3	80	18.19	148
IWW	69	1.3	144	15.96	213
SVV	70	1.3	4	21.73	74
SFW	71	1.3	85	18.02	156
AFT	72	1.3	83	18.03	155
AEI	73	1.3	78	17.97	151
AFX	74	1.3	82	18.09	156
CHA	75	1.3	86	17.98	161
GME	76	1.3	87	18.26	163
IWV	77	1.3	151	15.63	228

UOS	78	1.2	51	18.78	129
**Zeolite Framework**	**Rank of Adsorption Loading**	**Adsorption Loading**	**Rank of Adsorption Energy**	**Adsorption Energy (kcal/mol)**	**Sum of Rank**
CSV	79	1.2	108	17.39	187
UTL	80	1.2	153	15.6	233
EUO	81	1.2	23	19.87	104
SFG	82	1.2	41	19.09	123
SSF	83	1.2	96	17.7	179
SAV	84	1.2	162	15	246
SFE	85	1.2	115	17.2	200
RHO	86	1.2	16	17.07	102
RUT	87	1.2	118	20.24	205
SSY	88	1.2	128	16.71	216
ITR	89	1.2	22	19.9	111
VET	90	1.2	33	19.43	123
ITG	91	1.2	68	18.46	159
LTA	92	1.2	130	16.67	222
IHW	93	1.2	11	20.84	104
BOG	94	1.2	95	17.72	189
GON	95	1.2	46	18.88	141
IFW	96	1.1	131	16.62	227
SFF	97	1.1	43	19.06	140
OSI	98	1.1	44	18.9	142
MOZ	99	1.1	75	18.32	174
SFS	100	1.1	88	17.94	188
SGT	101	1.1	10	21.03	111
IFO	102	1.1	167	14.67	269
IFR	103	1.1	77	18.27	180
OWE	104	1.1	137	16.42	241
STT	105	1.1	37	19.21	142
TUN	106	1.1	66	18.48	172
STF	107	1.1	89	17.88	196
CFI	108	1	138	16.27	246
SFN	109	1	158	15.21	267
IRN	110	1	136	16.44	246
SEW	111	1	79	18.23	190
SFH	112	1	160	15.12	272
MER	113	1	21	19.95	134
DON	114	1	1	26.03	115
AET	115	1	135	16.53	250
RTH	116	1	100	17.6	216
MTN	117	1	20	19.99	137
EEI	118	1	18	20.07	136

ITE	119	1	120	17.06	239
**Zeolite Framework**	**Rank of Adsorption Loading**	**Adsorption Loading**	**Rank of Adsorption Energy**	**Adsorption Energy (kcal/mol)**	**Sum of Rank**
MFS	120	1	31	19.46	151
FER	121	1	45	18.88	166
LIO	122	1	111	14.39	233
UFI	123	1	169	17.33	292
SZR	124	0.9	106	17.44	230
AFR	125	0.9	165	14.83	290
MTF	126	0.9	5	21.66	131
SSO	127	0.9	124	16.89	251
ATT	128	0.9	164	14.91	292
SAS	129	0.9	143	16.15	272
ITH	130	0.9	117	17.11	247
AFO	131	0.9	29	19.62	160
ETL	132	0.9	14	20.54	146
MAZ	133	0.9	98	17.62	231
AEL	134	0.9	70	18.4	204
AFY	135	0.9	12	13.65	147
DDR	136	0.9	173	20.63	309
TER	137	0.9	81	18.11	218
EON	138	0.8	99	17.61	237
NON	139	0.8	19	20.04	158
MRE	140	0.8	133	16.56	273
SVR	141	0.8	84	18.03	225
IMF	142	0.8	67	18.46	209
MSO	143	0.8	91	17.83	234
ASV	144	0.8	168	14.52	312
MFI	145	0.8	32	19.44	177
IFY	146	0.7	55	18.64	201
MEL	147	0.7	103	17.57	250
PAU	148	0.7	52	18.72	200
GIU	149	0.7	101	17.59	250
MOR	150	0.7	116	17.16	266
AFI	151	0.7	152	15.61	303
SFV	152	0.7	102	17.58	254
STO	153	0.7	104	17.5	257
RON	154	0.6	49	18.83	203
SOF	155	0.6	94	17.73	249
LTF	156	0.6	132	16.57	288
DAC	157	0.6	142	16.17	299
SAF	158	0.6	126	16.86	284
LTL	159	0.6	129	16.69	288

STW	160	0.6	112	17.29	272
**Zeolite Framework**	**Rank of Adsorption Loading**	**Adsorption Loading**	**Rank of Adsorption Energy**	**Adsorption Energy (kcal/mol)**	**Sum of Rank**
AFN	161	0.5	36	19.31	197
HEU	162	0.5	114	17.22	276
DOH	163	0.5	48	18.84	211
FAR	164	0.4	97	17.63	261
JSW	165	0.4	27	19.7	192
PCR	166	0.4	59	18.58	225
LTN	167	0.3	145	15.88	312
MWF	168	0.3	50	18.82	218
ATO	169	0.3	174	12.6	343
SIV	170	0.3	72	18.35	242
TOL	171	0.2	172	13.73	343
SOS	172	0.2	156	15.39	328
JST	173	0.1	56	18.62	229
EPI	174	0.1	161	15.04	335
ABW	175	0	175	0	350
ACO	176	0	176	0	352
AEN	177	0	177	0	354
AHT	178	0	178	0	356
ANA	179	0	179	0	358
APC	180	0	180	0	360
APD	181	0	181	0	362
ATN	182	0	182	0	364
ATV	183	0	183	0	366
AWO	184	0	184	0	368
BCT	185	0	185	0	370
BIK	186	0	186	0	372
BOF	187	0	187	0	374
BRE	188	0	188	0	376
BSV	189	0	189	0	378
CAS	190	0	190	0	380
CDO	191	0	191	0	382
CGF	192	0	192	0	384
CHI	193	0	193	0	386
CZP	194	0	194	0	388
DFT	195	0	195	0	390
GIS	196	0	196	0	392
GOO	197	0	197	0	394
ITW	198	0	198	0	396
JBW	199	0	199	0	398
JNT	200	0	200	0	400

JOZ	201	0	201	0	402
**Zeolite Framework**	**Rank of Adsorption Loading**	**Adsorption Loading**	**Rank of Adsorption Energy**	**Adsorption Energy (kcal/mol)**	**Sum of Rank**
JRY	202	0	202	0	404
JSN	203	0	203	0	406
LIT	204	0	204	0	408
LOV	205	0	205	0	410
LTJ	206	0	206	0	412
MAR	207	0	207	0	414
MEP	208	0	208	0	416
MON	209	0	209	0	418
MVY	210	0	210	0	420
NAB	211	0	211	0	422
NAT	212	0	212	0	424
NPO	213	0	213	0	426
NSI	214	0	214	0	428
PAR	215	0	215	0	430
PON	216	0	216	0	432
PSI	217	0	217	0	434
RRO	218	0	218	0	436
RSN	219	0	219	0	438
RWR	220	0	220	0	440
SBN	221	0	221	0	442
SOD	222	0	222	0	444
THO	223	0	223	0	446
TON	224	0	224	0	448
UEI	225	0	225	0	450
UOZ	226	0	226	0	452
VNI	227	0	227	0	454
VSV	228	0	228	0	456
WEI	229	0	229	0	458
WEN	230	0	230	0	460
YUG	231	0	231	0	462
ZON	232	0	232	0	464

Table S3. The adsorption loading (molec nm^{-3}) and adsorption energy (kcal mol^{-1}) of all the frameworks toward DMSD.

Zeolite Framework	Rank of Adsorption Loading	Adsorption Loading	Rank of Adsorption Energy	Adsorption Energy (kcal/mol)	Sum of Rank
RWY	1	3.6	167	15.19	168
EDI	2	3.3	184	13.87	186
IRR	3	3.2	132	16.25	135
IRY	4	2.9	161	15.44	165
ITV	5	2.8	182	14.31	187
SOD	6	2.8	187	13.51	193
NPT	7	2.6	43	18.12	50
CLO	8	2.6	178	14.79	186
IFU	9	2.5	157	15.5	166
OBW	10	2.4	109	16.63	119
SBS	11	2.3	73	17.3	84
SAO	12	2.3	80	17.23	92
AVL	13	2.3	35	18.33	48
SBT	14	2.2	100	16.77	114
ETR	15	2.2	26	18.57	41
ITT	16	2.2	183	14.21	199
_CON	17	2.2	8	19.35	25
MEI	18	2.2	96	16.79	114
EWT	19	2.1	102	16.75	121
OWE	20	2.1	46	18	66
EMT	21	2.1	135	16.14	156
CGS	22	2.1	25	18.62	47
JSR	23	2.1	171	14.98	194
BPH	24	2.1	88	17.06	112
AFS	25	2.1	130	16.26	155
FRA	26	2.1	126	16.37	152
AFV	27	2.1	11	19.23	38
VFI	28	2	139	16.06	167
ZON	29	2	15	18.99	44
DFO	30	2	68	17.54	98
BRE	31	2	45	18.03	76
IWS	32	2	93	16.87	125
UOV	33	2	19	18.74	52

Zeolite Framework	Rank of Adsorption Loading	Adsorption Loading	Rank of Adsorption Energy	Adsorption Energy (kcal/mol)	Sum of Rank
IWR	34	2	55	17.86	89
PHI	35	2	60	17.74	95
LTA	36	1.9	74	17.29	110
POS	37	1.9	79	17.23	116
BEA	38	1.9	94	16.8	132
SBE	39	1.9	147	15.81	186
AFR	40	1.9	117	16.53	157
SFO	41	1.9	124	16.41	165
BEC	42	1.9	118	16.52	160
ISV	43	1.9	115	16.55	158
FAU	44	1.9	163	15.39	207
STI	45	1.9	16	18.96	61
SEW	46	1.8	27	18.54	73
RUT	47	1.8	4	20.08	51
SAV	48	1.8	170	15.13	218
SVV	49	1.8	5	19.95	54
OFF	50	1.8	113	16.58	163
ERI	51	1.8	34	18.39	85
ITG	52	1.8	12	19.15	64
EAB	53	1.8	65	17.61	118
SFS	54	1.8	51	17.91	105
LEV	55	1.8	123	16.43	178
AFY	56	1.8	168	15.17	224
MSE	57	1.7	31	18.44	88
BOG	58	1.7	56	17.84	114
ITN	59	1.7	86	17.12	145
IWW	60	1.7	129	16.34	189
IFO	61	1.7	146	15.82	207
UFI	62	1.7	70	17.4	132
BOZ	63	1.7	164	15.32	227
FER	64	1.7	77	17.25	141
MAZ	65	1.7	85	17.13	150
IFR	66	1.7	21	18.69	87
PUN	67	1.7	82	17.22	149
RHO	68	1.7	121	16.45	189
ASV	69	1.7	10	19.26	79
TSC	70	1.6	2	21.46	72

Zeolite Framework	Rank of Adsorption Loading	Adsorption Loading	Rank of Adsorption Energy	Adsorption Energy (kcal/mol)	Sum of Rank
UTL	71	1.6	116	16.54	187
KFI	72	1.6	87	17.09	159
CSV	73	1.6	54	17.89	127
USI	74	1.6	49	17.95	123
JST	75	1.6	72	17.37	147
AST	76	1.6	33	18.42	109
IWV	77	1.6	131	16.25	208
SSO	78	1.6	29	18.47	107
ATO	79	1.6	150	15.71	229
OKO	80	1.5	52	17.91	132
NES	81	1.5	48	17.97	129
MWW	82	1.5	89	17	171
THO	83	1.5	162	15.44	245
JSW	84	1.5	13	19.14	97
MTT	85	1.5	133	16.21	218
TON	86	1.5	174	14.91	260
LAU	87	1.5	42	18.13	129
UWY	88	1.5	125	16.4	213
BOF	89	1.5	9	19.33	98
OSO	90	1.5	185	13.74	275
ESV	91	1.5	3	20.49	94
IRN	92	1.5	127	16.36	219
SOF	93	1.4	108	16.65	201
DAC	94	1.4	104	16.71	198
EZT	95	1.4	50	17.92	145
RTE	96	1.4	22	18.69	118
ATT	97	1.4	181	14.33	278
SVR	98	1.4	47	18	145
UOS	99	1.4	58	17.83	157
AFG	100	1.4	18	18.92	118
AWW	101	1.4	23	18.64	124
AFI	102	1.4	40	18.16	142
CAN	103	1.4	142	15.98	245
CDO	104	1.4	158	15.47	262
LOS	105	1.4	32	18.44	137
SAS	106	1.4	99	16.78	205
SSF	107	1.4	97	16.79	204

Zeolite Framework	Rank of Adsorption Loading	Adsorption Loading	Rank of Adsorption Energy	Adsorption Energy (kcal/mol)	Sum of Rank
LIO	108	1.4	186	13.68	294
HEU	109	1.4	6	18.19	115
UOZ	110	1.4	38	19.81	148
SAT	111	1.4	75	17.27	186
IFW	112	1.4	148	15.76	260
SFG	113	1.4	64	17.63	177
LTF	114	1.3	111	16.6	225
ATS	115	1.3	138	16.07	253
GIU	116	1.3	106	16.68	222
IHW	117	1.3	17	18.94	134
MAR	118	1.3	176	14.86	294
MTW	119	1.3	44	18.04	163
SFW	120	1.3	134	16.14	254
AFT	121	1.3	149	15.75	270
AEI	122	1.3	59	15.53	181
AFX	123	1.3	145	15.83	268
CHA	124	1.3	152	15.69	276
GME	125	1.3	155	17.82	280
TUN	126	1.3	81	17.23	207
ITH	127	1.2	78	17.25	205
ITR	128	1.2	24	18.63	152
MOZ	129	1.2	90	17	219
STW	130	1.2	110	16.63	240
EUO	131	1.2	71	17.37	202
STT	132	1.2	114	16.56	246
SFE	133	1.2	137	16.1	270
SSY	134	1.2	112	16.59	246
TOL	135	1.2	136	16.13	271
MSO	136	1.2	36	18.22	172
VET	137	1.2	37	18.22	174
GON	138	1.2	57	17.83	195
SOS	139	1.1	172	14.94	311
EON	140	1.1	173	14.94	313
SFF	141	1.1	120	16.48	261
OSI	142	1.1	76	17.26	218
EPI	143	1.1	153	15.66	296
SGT	144	1.1	41	18.15	185

Zeolite Framework	Rank of Adsorption Loading	Adsorption Loading	Rank of Adsorption Energy	Adsorption Energy (kcal/mol)	Sum of Rank
DON	145	1.1	1	22.91	146
EEI	146	1.1	30	18.46	176
IMF	147	1.1	101	16.76	248
STF	148	1.1	159	15.45	307
CFI	149	1	169	15.16	318
AFN	150	1	61	17.68	211
RTH	151	1	166	15.31	317
SFN	152	1	179	14.54	331
SFH	153	1	180	14.46	333
MER	154	1	62	17.65	216
AET	155	1	143	15.93	298
MTN	156	1	20	18.71	176
ETL	157	1	28	18.53	185
SIV	158	1	154	15.64	312
TER	159	1	144	15.9	303
ATN	160	1	165	15.32	325
IFY	161	1	14	19.05	175
ITE	162	1	177	14.79	339
SZR	163	1	122	16.43	285
MFS	164	1	98	16.78	262
AEL	165	1	39	18.17	204
MTF	166	0.9	7	19.39	173
AFO	167	0.9	66	17.58	233
FAR	168	0.9	84	17.19	252
DDR	169	0.9	53	17.9	222
SAF	170	0.9	141	16.04	311
PAU	171	0.9	107	16.67	278
CGF	172	0.8	151	15.7	323
SFV	173	0.8	140	16.05	313
MRE	174	0.8	91	16.98	265
NON	175	0.8	63	17.63	238
LTN	176	0.8	105	16.69	281
MFI	177	0.8	92	16.98	269
MEL	178	0.7	160	15.45	338
STO	179	0.7	156	15.5	335
MOR	180	0.7	175	14.91	355
LTL	181	0.7	103	16.71	284

Zeolite Framework	Rank of Adsorption Loading	Adsorption Loading	Rank of Adsorption Energy	Adsorption Energy (kcal/mol)	Sum of Rank
RON	182	0.7	119	16.49	301
GIS	183	0.6	191	11.44	374
PCR	184	0.6	69	17.41	253
PSI	185	0.6	67	17.54	252
MWF	186	0.5	95	16.79	281
DOH	187	0.5	128	16.36	315
WEN	188	0.4	189	11.92	377
MEP	189	0.4	190	11.91	379
PON	190	0.3	83	17.22	273
UEI	191	0.3	188	13.48	379
ABW	192	0	192	0	384
ACO	193	0	193	0	386
AEN	194	0	194	0	388
AHT	195	0	195	0	390
ANA	196	0	196	0	392
APC	197	0	197	0	394
APD	198	0	198	0	396
ATV	199	0	199	0	398
AWO	200	0	200	0	400
BCT	201	0	201	0	402
BIK	202	0	202	0	404
BSV	203	0	203	0	406
CAS	204	0	204	0	408
CHI	205	0	205	0	410
CZP	206	0	206	0	412
DFT	207	0	207	0	414
GOO	208	0	208	0	416
ITW	209	0	209	0	418
JBW	210	0	210	0	420
JNT	211	0	211	0	422
JOZ	212	0	212	0	424
JRY	213	0	213	0	426
JSN	214	0	214	0	428
LIT	215	0	215	0	430
LOV	216	0	216	0	432
LTJ	217	0	217	0	434
MON	218	0	218	0	436

Zeolite Framework	Rank of Adsorption Loading	Adsorption Loading	Rank of Adsorption Energy	Adsorption Energy (kcal/mol)	Sum of Rank
MVY	219	0	219	0	438
NAB	220	0	220	0	440
NAT	221	0	221	0	442
NPO	222	0	222	0	444
NSI	223	0	223	0	446
PAR	224	0	224	0	448
RRO	225	0	225	0	450
RSN	226	0	226	0	452
RWR	227	0	227	0	454
SBN	228	0	228	0	456
VNI	229	0	229	0	458
VSV	230	0	230	0	460
WEI	231	0	231	0	462
YUG	232	0	232	0	464

Table S4. The adsorption loading (molec nm^{-3}) and adsorption energy (kcal mol^{-1}) of all the frameworks toward MMST.

Zeolite Framework	Rank of Adsorption Loading	Adsorption Loading	Rank of Adsorption Energy	Adsorption Energy (kcal/mol)	Sum of Rank
RWY	1	4.2	81	16.7	82
IRY	2	3.6	75	16.79	77
IRR	3	3.5	53	17.15	56
MEI	4	3.5	2	19.73	6
AFY	5	3.4	8	18.89	13
ITV	6	3.3	146	15.44	152
JSR	7	3.3	70	16.83	77
EDI	8	3.3	196	13.64	204
PUN	9	3.2	5	19.53	14
THO	10	3.1	136	15.7	146
CLO	11	3	162	15.2	173
JOZ	12	3	130	15.81	142
IFU	13	3	102	16.34	115
NPT	14	2.9	51	17.21	65

Zeolite Framework	Rank of Adsorption Loading	Adsorption Loading	Rank of Adsorption Energy	Adsorption Energy (kcal/mol)	Sum of Rank
OBW	15	2.9	92	16.6	107
SBS	16	2.8	24	17.77	40
LTA	17	2.8	121	16	138
SOD	18	2.8	151	15.35	169
BEC	19	2.8	11	18.68	30
SBN	20	2.7	178	14.62	198
OSO	21	2.7	39	15.27	60
SBT	22	2.7	157	17.38	179
EWT	23	2.7	55	17.13	78
ITT	24	2.7	171	15.03	195
SAO	25	2.7	27	17.61	52
UFI	26	2.6	83	16.69	109
EMT	27	2.6	36	17.41	63
BPH	28	2.6	10	18.73	38
FAU	29	2.6	140	15.63	169
ETR	30	2.6	6	19.07	36
IWS	31	2.5	41	17.36	72
DFO	32	2.5	56	17.1	88
POS	33	2.4	42	17.35	75
VFI	34	2.4	33	17.44	67
RHO	35	2.4	61	17.03	96
WEN	36	2.4	183	14.49	219
SBE	37	2.4	100	16.35	137
ISV	38	2.4	32	17.45	70
CSV	39	2.4	17	18.04	56
BEA	40	2.3	46	17.31	86
BOZ	41	2.3	134	15.76	175
SFS	42	2.3	19	17.9	61
AFS	43	2.3	76	16.76	119
JST	44	2.3	135	15.74	179
BRE	45	2.3	25	17.72	70
UTL	46	2.3	45	17.31	91
AVL	47	2.3	72	16.81	119
ZON	48	2.3	49	17.3	97
_CON	49	2.2	48	17.3	97
IWR	50	2.2	38	17.38	88
CHA	51	2.2	113	16.21	164

Zeolite Framework	Rank of Adsorption Loading	Adsorption Loading	Rank of Adsorption Energy	Adsorption Energy (kcal/mol)	Sum of Rank
ATN	52	2.2	138	15.64	190
KFI	53	2.2	94	16.51	147
IFO	54	2.2	88	16.63	142
IFR	55	2.1	1	20.07	56
OWE	56	2.1	77	16.76	133
MEP	57	2.1	191	14.19	248
CGS	58	2.1	20	17.89	78
BOG	59	2.1	30	17.48	89
SAV	60	2.1	177	14.8	237
AFV	61	2.1	66	16.94	127
FRA	62	2.1	137	15.68	199
IWW	63	2.1	109	16.25	172
SFN	64	2.1	63	16.96	127
SEW	65	2.1	9	18.81	74
ITN	66	2.1	79	16.74	145
OKO	67	2.1	16	18.2	83
SAT	68	2.1	12	18.48	80
SFH	69	2	97	16.4	166
PHI	70	2	57	17.09	127
MSE	71	2	23	17.8	94
IRN	72	2	93	16.59	165
UOV	73	2	15	17.37	88
USI	74	2	40	18.27	114
FAR	75	2	126	15.93	201
IWV	76	2	7	16.21	83
RUT	77	2	112	19.04	189
AFT	78	2	87	16.65	165
UWY	79	2	104	16.34	183
MAR	80	2	118	16.13	198
CDO	81	2	62	17.02	143
AFX	82	2	65	16.95	147
FER	83	2	101	16.35	184
HEU	84	1.9	90	16.6	174
SVV	85	1.9	14	18.43	99
JSN	86	1.9	141	15.62	227
UOZ	87	1.9	21	17.88	108
ASV	88	1.9	74	16.8	162

Zeolite Framework	Rank of Adsorption Loading	Adsorption Loading	Rank of Adsorption Energy	Adsorption Energy (kcal/mol)	Sum of Rank
AFR	89	1.9	122	15.09	211
SFO	90	1.9	169	15.98	259
EZT	91	1.9	13	18.47	104
TSC	92	1.9	3	19.63	95
SAS	93	1.9	71	16.81	164
STI	94	1.9	78	16.75	172
ATS	95	1.9	69	15.88	164
MAZ	96	1.9	127	16.87	223
SSF	97	1.8	26	17.64	123
RTH	98	1.8	96	16.44	194
GIS	99	1.8	195	13.99	294
OFF	100	1.8	180	14.53	280
ERI	101	1.8	103	16.34	204
GME	102	1.8	181	14.51	283
ITG	103	1.8	47	17.3	150
EAB	104	1.8	145	15.49	249
LEV	105	1.8	194	14.09	299
MEL	106	1.8	99	16.35	205
GIU	107	1.8	91	16.6	198
NES	108	1.7	37	17.4	145
MWW	109	1.7	95	16.49	204
ITE	110	1.7	120	16.06	230
SSO	111	1.7	85	16.68	196
SIV	112	1.7	143	15.56	255
SFW	113	1.6	110	16.23	223
SOF	114	1.6	168	15.03	282
STW	115	1.6	172	15.1	287
AEI	116	1.6	187	14.28	303
IFW	117	1.6	158	15.27	275
SFF	118	1.6	149	15.41	267
STT	119	1.6	58	17.06	177
LTF	120	1.6	115	16.16	235
EPI	121	1.6	159	15.25	280
AST	122	1.6	142	15.58	264
STF	123	1.6	192	14.11	315
ATO	124	1.6	67	16.92	191
ITH	125	1.5	44	17.32	169

Zeolite Framework	Rank of Adsorption Loading	Adsorption Loading	Rank of Adsorption Energy	Adsorption Energy (kcal/mol)	Sum of Rank
LTN	126	1.5	111	16.22	237
JSW	127	1.5	59	17.05	186
BOF	128	1.5	86	16.65	214
ITR	129	1.5	34	17.44	163
MTT	130	1.5	52	17.18	182
TON	131	1.5	105	16.32	236
LAU	132	1.5	116	16.15	248
TUN	133	1.5	119	16.06	252
SVR	134	1.5	98	16.36	232
EON	135	1.5	82	16.69	217
TER	136	1.5	125	15.94	261
ESV	137	1.5	22	17.87	159
LIO	138	1.5	139	15.64	277
IMF	139	1.5	132	15.79	271
UOS	140	1.5	148	15.43	288
MSO	141	1.5	54	17.14	195
UEI	142	1.5	174	14.96	316
DAC	143	1.5	144	15.52	287
IFY	144	1.5	29	17.51	173
RTE	145	1.4	64	15.95	209
SFG	146	1.4	124	16.95	270
ATT	147	1.4	147	15.44	294
SOS	148	1.4	193	14.09	341
AFG	149	1.4	89	16.61	238
AWW	150	1.4	131	15.81	281
AFI	151	1.4	50	17.24	201
CAN	152	1.4	129	15.86	281
LOS	153	1.4	107	16.27	260
TOL	154	1.4	114	16.16	268
MOZ	155	1.4	128	15.87	283
EUO	156	1.3	106	16.3	262
IHW	157	1.3	28	17.51	185
PON	158	1.3	176	14.91	334
MTW	159	1.3	133	15.79	292
AFN	160	1.3	163	15.18	323
SSY	161	1.3	182	14.5	343
STO	162	1.3	43	17.32	205

Zeolite Framework	Rank of Adsorption Loading	Adsorption Loading	Rank of Adsorption Energy	Adsorption Energy (kcal/mol)	Sum of Rank
SFE	163	1.2	186	14.3	349
SAF	164	1.2	35	17.43	199
VET	165	1.2	68	16.9	233
GON	166	1.2	73	16.8	239
MOR	167	1.2	18	17.95	185
PAU	168	1.2	170	15.04	338
SFV	169	1.1	160	15.23	329
EEI	170	1.1	60	17.04	230
CGF	171	1.1	117	16.15	288
MFI	172	1.1	152	15.34	324
OSI	173	1.1	153	15.31	326
SGT	174	1.1	155	15.29	329
DON	175	1.1	4	19.59	179
CFI	176	1	197	13.39	373
MER	177	1	164	15.17	341
AET	178	1	165	15.16	343
MTN	179	1	80	16.71	259
ETL	180	1	108	16.26	288
SZR	181	1	188	14.28	369
MFS	182	1	189	14.27	371
AFO	183	1	150	15.36	333
AEL	184	1	154	15.3	338
MTF	185	0.9	84	16.68	269
PCR	186	0.9	156	15.27	342
DDR	187	0.9	161	15.23	348
ITW	188	0.9	184	14.4	372
MWF	189	0.8	175	14.94	364
MRE	190	0.8	166	15.12	356
NON	191	0.8	167	15.11	358
LTL	192	0.8	31	17.46	223
RON	193	0.8	173	14.98	366
AWO	194	0.7	200	12.24	394
BSV	195	0.6	179	14.58	374
RRO	196	0.6	198	13.32	394
PSI	197	0.6	123	15.98	320
DOH	198	0.6	185	14.38	383
NAT	199	0.4	203	10.7	402

Zeolite Framework	Rank of Adsorption Loading	Adsorption Loading	Rank of Adsorption Energy	Adsorption Energy (kcal/mol)	Sum of Rank
YUG	200	0.4	199	12.99	399
VNI	201	0.3	190	14.26	391
GOO	202	0.2	202	11.54	404
JRY	203	0.2	201	11.56	404
ABW	204	0	204	0	408
ACO	205	0	205	0	410
AEN	206	0	206	0	412
AHT	207	0	207	0	414
ANA	208	0	208	0	416
APC	209	0	209	0	418
APD	210	0	210	0	420
ATV	211	0	211	0	422
BCT	212	0	212	0	424
BIK	213	0	213	0	426
CAS	214	0	214	0	428
CHI	215	0	215	0	430
CZP	216	0	216	0	432
DFT	217	0	217	0	434
JBW	218	0	218	0	436
JNT	219	0	219	0	438
LIT	220	0	220	0	440
LOV	221	0	221	0	442
LTJ	222	0	222	0	444
MON	223	0	223	0	446
MVY	224	0	224	0	448
NAB	225	0	225	0	450
NPO	226	0	226	0	452
NSI	227	0	227	0	454
PAR	228	0	228	0	456
RSN	229	0	229	0	458
RWR	230	0	230	0	460
VSV	231	0	231	0	462

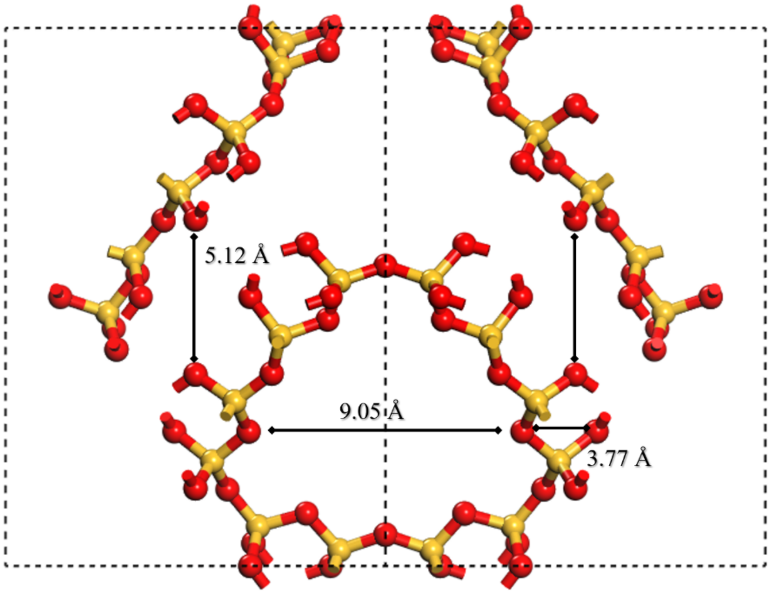

Figure S1. Side view of RWY zeolite framework.
